# Development of a one-pot assay for screening and identification of Mur pathway inhibitors in *Mycobacterium tuberculosis*

**DOI:** 10.1038/srep35134

**Published:** 2016-10-13

**Authors:** Kandasamy Eniyan, Anuradha Kumar, Geetha Vani Rayasam, Andrej Perdih, Urmi Bajpai

**Affiliations:** 1 Department of Biomedical Science, Acharya Narendra Dev College, University of Delhi, New Delhi 110019, India; 2 Infectious Disease Research Institute, Seattle WA 98102, USA; 3 CSIR-IGIB, South Campus, Mathura road, New Delhi 110025, India; 4 National Institute of Chemistry, Hajdrihova 19, 1001 Ljubljana, Slovenia

## Abstract

The cell wall of *Mycobacterium tuberculosis* (Mtb) consists of peptidoglycan, arabinogalactan and mycolic acids. The cytoplasmic steps in the peptidoglycan biosynthetic pathway, catalyzed by the Mur (A-F) enzymes, involve the synthesis of UDP-n-acetylmuramyl pentapeptide, a key precursor molecule required for the formation of the peptidoglycan monomeric building blocks. Mur enzymes are indispensable for cell integrity and their lack of counterparts in eukaryotes suggests them to be promising Mtb drug targets. However, the caveat is that most of the current assays utilize a single Mur enzyme, thereby identifying inhibitors against only one of the enzymes. Here, we report development of a one-pot assay that reconstructs the entire Mtb Mur pathway *in vitro* and has the advantage of eliminating the requirement for nucleotide intermediates in the pathway as substrates. The MurA-MurF enzymes were purified and a one-pot assay was developed through optimization of successive coupled enzyme assays using UDP-n-acetylglucosamine as the initial sugar substrate. The assay is biochemically characterized and optimized for high-throughput screening of molecules that could disrupt multiple targets within the pathway. Furthermore, we have validated the assay by performing it to identify D-Cycloserine and furan-based benzene-derived compounds with known Mur ligase inhibition as inhibitors of Mtb MurE and MurF.

Tuberculosis (TB) is a major health concern and worldwide emergence of multi-drug resistant and extensively-drug resistant forms of *Mycobacterium tuberculosis* (Mtb) has further aggravated the situation^1^,^2^,^3^. Most of the available clinical drugs have single targets in the pathogens and hence are very often rendered ineffective over a period of time due to the development of the bacterial resistance against them. Novel strategies are needed for finding new anti-Mtb drugs, which should address resistance mechanisms wherein a single point mutation in the active site of the target protein leaves the antibiotic ineffective, as observed in the case of resistance of Mtb MurA against fosfomycin^4^,^5^. Therefore, not only are new TB drugs required to combat the menace of antibiotic resistance, alternative approaches for finding new drugs are also important. One such approach that will have obvious advantages in clinical use is a multi-targeted therapy, where a single drug targets multiple enzymes in key metabolic pathways of the pathogen and hence reduces the probability of occurrence of drug-resistance^6^,^7^,^8^.

Mtb encodes a series of pathways that are (i) unique to the bacterium, (ii) essential for its growth, (iii) absent in mammalian cells and hence are promising targets for the selective inhibition of pathogen growth while sparing the host with potentially reduced side effects^8^,^9^,^10^. The cell wall of Mtb is one such target, which is composed of three covalently linked macromolecules: peptidoglycan, arabinogalactan and mycolic acids^11^,^12^,^13^. The peptidoglycan biosynthesis is a complex process that involves two stages: cytoplasmic and in membrane. The cytoplasmic steps in the biosynthetic pathway of peptidoglycan involves the synthesis of UDP-n-acetylmuramyl pentapeptide (UDP-MurNAc-pentapeptide), catalyzed by Mur enzymes (MurA-MurF) (Fig. 1). The soluble UDP-MurNAc-pentapeptide is then translocated to the periplasmic space, where the penicillin binding proteins (PBPs) carry out transglycosylation and transpeptidation reactions which result in the synthesis of mature peptidoglycan^14^,^15^. Mur enzymes are highly conserved amongst bacteria, have no counterparts in eukaryotes and are essential for maintaining cell integrity and for resistance to variations in osmotic pressure^12^,^13^,^16^.

Amongst the Mur pathway enzymes, MurC-MurF (ATP-dependent ligases) are closely homologous to each other but MurA, a transferase and MurB a reductase are distinctly different from Mur ligases as well from each other. The first step in the Mur pathway is catalyzed by MurA where the enolypyruvate moiety from phosphoenolpyruvate (PEP) is transferred to UDP-n-acetylglucosamine (UDP-GlcNAc). Subsequently, MurB reduces the enolpyruvate moiety to a lactyl group using NADPH as the cofactor and forms UDP-n-acetylmuramic acid (UDP-MurNAc). In the next four steps, Mur ligases (MurC-MurF) catalyze the sequential addition of amino acids L-Alanine, D-Glutamate, *meso*-Diaminopimelic acid and D-Alanine-D-Alanine to UDP-MurNAc to form UDP-MurNAc pentapeptide. Though Mur ligases have been characterized in several micro-organisms^12^,^17^, knowledge of the structure, function and regulation of these enzymes in Mtb has largely been fragmented^18^,^19^,^20^. Recently a significant study^21^ has characterized the *dcw* operon in Mtb and has provided deeper insights into the function and regulatory network of Mur ligase enzymes. Mur ligases possess several conserved amino acid residues in their active sites for substrate binding and enzyme activity, act using the same catalytic mechanism, and share key structural features. They proceed via an ordered kinetic mechanism with a sequential substrate binding, beginning with ATP, followed by the nucleotide substrate and ending with the amino acid or dipeptide^12^,^22^,^23^. Each Mur ligase is composed of an N-terminal domain which binds the nucleotide substrate, a central domain which binds with ATP and a C-terminal domain that binds with the amino acid substrates^12^,^24^. Though the Mtb Mur ligases do not show significant sequence identity, however their ATP binding sites are found to be highly conserved.

While the Mur pathway in other pathogens has been exploited in the past for developing inhibitors^25^,^26^,^27^,^28^,^29^, fewer such studies^30^,^31^,^32^,^33^ have been carried out on the Mur enzymes from Mtb where inhibitors have been screened using a single Mur enzyme as the target (leading to identification of inhibitors for only one enzyme at a time). This method not only makes the screening of inhibitors time consuming, laborious and expensive, but also has an inherent potential for finding drugs against which the pathogen could develop resistance in a rather short time. A one-pot assay is an assay that reconstructs the entire Mur pathway *in vitro*, offers the advantage of facilitating the screening of molecules that disrupt multiple targets within the pathway.

In our study, through the one-pot assay, UDP-MurNAc pentapeptide was synthesized as the final product by including the initial substrates UDP-n-acetylglucosamine (UDP-GlcNAc) and Phosphoenolpyruvate (PEP), all six Mur enzymes and cofactors together. Here the product formed in the first reaction of the pathway serves as the substrate for the next enzyme and so forth. This eliminates the requirement for nucleotide intermediates in the Mur pathway as substrates, which in general are not widely available commercially. In addition, it keeps the cost of the assay and that of high throughput screening low. So far, only two groups have demonstrated the development of a one-pot assay with Mur enzyme (A-F): from *E.coli*^34^ and from *P.aeruginosa*^35^. However, the assays developed in these studies have used large reaction volumes and long period of incubation, which are not optimal for high throughput screening (HTS). Hence, the focus of this study was to purify all six Mtb Mur enzymes (MurA-MurF) and develop a one-pot assay that could be used for screening of a large number of inhibitors molecules in a single assay.

## Results and Discussion

Currently available anti-mycobacterial cell-wall inhibitors inhibit enzymes involved in the later stage of peptidoglycan synthesis^36^,^37^. The Mur enzymes participate in the initial, intracellular steps of the same pathway and inhibitors specific to them remain unexplored. Hence, there is a clear opportunity to discover new molecules against the Mtb Mur enzymes and develop them into novel drug entities. Since the Mur ligases share highly conserved residues in their active sites and their mode of action is similar as corroborated by various studies^12^,^38^,^39^,^40^, they are amenable to multi-target therapy. Furthermore, these enzymes proceed via the same catalytic mechanism^12^,^22^,^23^,^24^ and provide an opportunity for the rational design and/or structure-based optimization of inhibitors for the development of molecules that efficiently target multiple Mur ligases. Therefore, to find new inhibitors against these enzymes, we have developed and validated a HTS-amenable one-pot assay that offers huge potential to screen and identify inhibitors that target more than one enzyme in the Mtb Mur pathway.

### Purification of Mur enzymes

The *murA*, *murB*, *murC*, *murD*, *murE* and *murF* genes of Mtb were cloned into the pET28a vector for expression as-N-terminal His-tagged proteins in *E. coli* BL21 (DE3). Purification of the Mur enzymes involved extensive optimization, as they formed insoluble inclusion bodies. Replacing *E. coli* BL21 (DE3) with *E. coli* C41 (DE3) significantly enhanced solubility of MurA-C and MurE. However, MurD and MurF could not be obtained in a soluble form under variable conditions of temperature, period of incubation, concentration of inducer, etc. Hence, MurD and MurF were cloned in another expression vector pMALc2x, containing an N-terminal maltose binding protein (MBP) tag and expressed in *E.coli* Rosetta-gami™ (DE3) pLysS, which yielded the proteins in the soluble fraction. Rosetta-gami™ host strains combine the advantages of Rosetta 2 and Origami 2 strains to alleviate codon bias and enhance disulfide bond formation in the cytoplasm when heterologous proteins are expressed in *E.coli*. These strains supply tRNAs for the rare codons on a compatible chloramphenicol-resistant plasmid. The purity obtained for each enzyme was above (90%) as analyzed by SDS-PAGE (Fig. 2).

### Development and optimization of Mur enzyme assays

**Figure 1:**
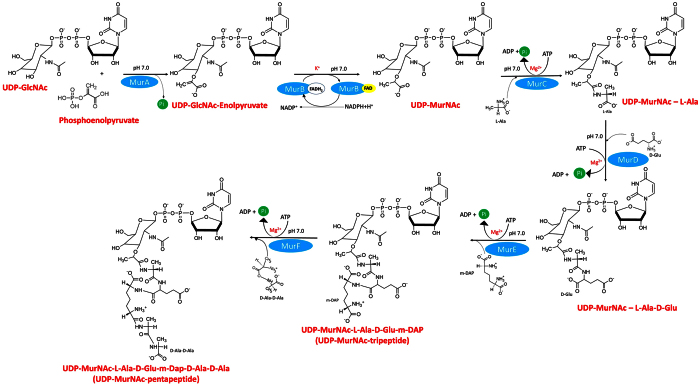
Schematic representation of the coupled assays for the Mur enzymes performed to reconstruct the Mur pathway *in vitro*. Incubation temperature was kept at 37 °C throughout the experiment.

**Figure 2:**
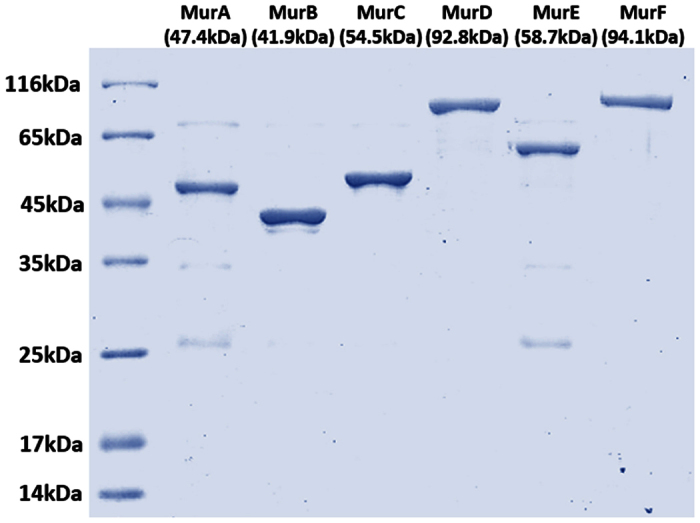
SDS-PAGE analysis of the purified recombinant Mtb Mur enzymes (MurA-MurF). Protein molecular weight markers (Lane 1); MurA-MurF (Lane 2-Lane 7). The molecular weight of MurD and MurF includes its MBP tag (42.5 kDa) along with their actual molecular weight (50.3 kDa and 51.6 kDa, respectively). 1 μg of total proteins was loaded in the gel.

Setting up the reactions to reconstruct the Mur pathway i*n vitro*, with multiple enzymes, substrates and cofactors represented a complex and challenging task. The assays were carried out sequentially with the initial assay involving the MurA enzyme and its substrates UDP-GlcNAc and PEP. The reaction product of MurA served as the substrate of MurB and the MurB reaction was initiated by addition of MurB, NADPH and KCl. This was followed sequentially by the addition of the MurC-MurF enzymes, MgCl_2_, ATP and L-Alanine, D-Glutamate, *meso*-Diaminopimelic acid, D-Alanine-D-Alanine as the respective substrate for each enzyme, resulting in the final product UDP-MurNAc pentapeptide (Fig. 1).

MurA, the first enzyme of the pathway was the only enzyme that was provided with its substrates (PEP and UDP-GlcNAc), that did not require addition of the cofactors in the assay and for which steady state kinetic studies were performed. The assay for MurA was an end point assay and its activity (nmol P_i_/min) was measured as the net P_i_ released from PEP. Prior to initiation of MurA enzyme reaction, MurA was pre-incubated with UDP-GlcNAc for 15 minutes. This step was included since UDP-MurNAc has been shown^41^,^42^ to co-purify with recombinant MurA isolated from *E.coli* and form a stable complex. This complex can be disassociated by its incubation with either UDP-GlcNAc (the substrate) or with P_i_ (the product). Hence, while setting up the reaction, we found pre-incubation of MurA to be a necessary step since its activity was observed to be significantly lower in the absence of pre-incubation (data not shown).

**Figure 3:**
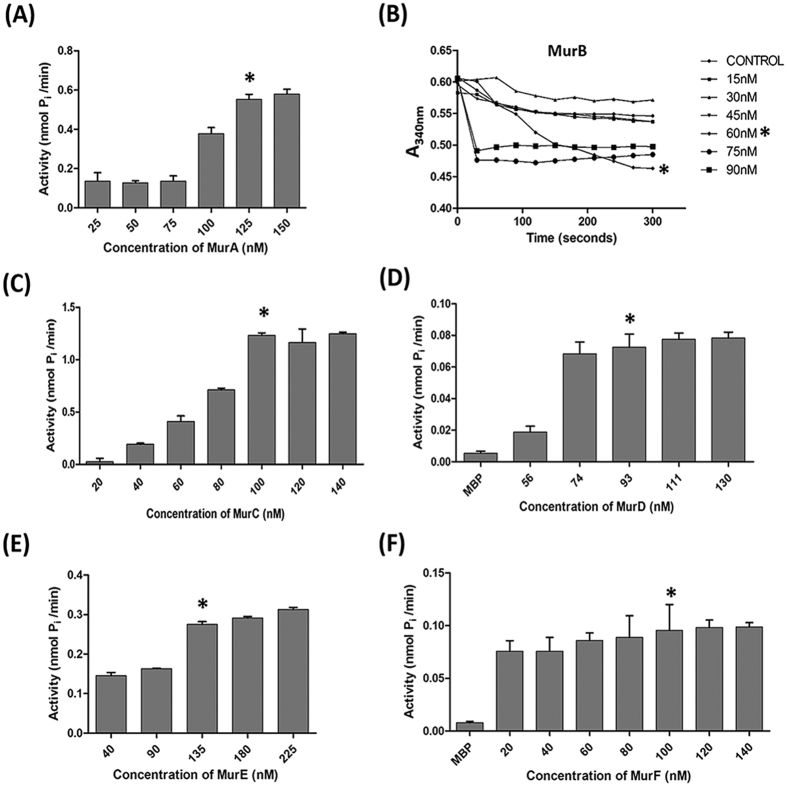
Activities of the Mur enzymes (MurA-MurF) at various concentrations. **(A)** Activity of MurA. The reaction contained 0.2 mM UDP-GlcNAc, 0.2 mM PEP and MurA enzyme at various concentrations. The X-axis represents the concentration of MurA enzyme (nM) and the Y-axis represents net P_i_ (nmol P_i_/min). **(B)** Activity of MurB. The MurA reaction after a 30 min of incubation time was supplemented with 10 mM KCl, 0.2 mM NADPH and MurB enzyme at various concentrations in a total volume of 100 μl. The absorbance at 340 nm was monitored over 5 min to measure the rate of NADPH oxidation. The X-axis represents incubation time (seconds) and the Y-axis represents absorbance at 340 nm. **(C–F)** Activity of Mur ligases. The MurB reaction after a 5 min of incubation time was supplemented with 10 mM MgCl_2_, 1 mM L-Alanine, 2 mM ATP and MurC enzyme at various concentrations. The reaction was incubated at 37 °C for 60 minutes. Likewise for MurD assay, MurC reaction was supplemented with 1 mM D-Glutamate and MurD enzyme, for MurE assay MurD reaction was supplemented with 1 mM *meso*-Diaminopimelic acid and MurE enzyme and for MurF assay, MurE reaction was supplemented with 1 mM D-Alanine-D-Alanine and MurF enzyme. (**C**) MurC, (**D**) MurD (**E**) MurE (**F**) MurF. The X-axis represents a Mur ligases at various concentrations (nM) and the Y-axis represents net P_i_ (nmol P_i_/min). On the bar/graph, “*” indicates the concentration of enzyme that was selected to be used subsequently in the respective assays. Data depicted is the mean ± S.E. values obtained from three independent experiments.

The MurB assay was kinetic and the enzyme activity was measured in terms of the rate of oxidation of NADPH (Fig. 3B). Initially, MurB activity was measured for 10 minutes and was found to reach plateau after 5 minutes of incubation and hence the activity was recorded up to 5 minutes only. The assay for Mur ligases were also an end point and their activity (nmol P_i_/min) was measured as the net P_i_ released from ATP.

Appropriate buffer conditions for the assay were analysed by testing various buffers at different pH values. For MurA to MurC, a HEPES buffer at pH 7.0 was found to be optimal. However, MurD-MurF showed poor activity in HEPES buffer but demonstrated improved results on changing the buffer to Bis-tris propane (pH 7.0). Hence Bis-tris propane was used for the subsequent experiments, as it was compatible for assaying all the Mur enzymes (Fig. S1).

In order to optimize the concentration of Mur enzymes to be added in the assays, each enzyme was assayed at its various concentrations (nM). The concentration at which enzyme activity was found to plateau was selected for the subsequent assays (Fig. 3).

### Optimization of substrates and cofactors

While setting up enzyme reactions, we used the concentration of the substrates and cofactors described in earlier reports on Mur enzymes^21^,^27^,^34^,^43^ as the starting point and then further optimization was carried out. The kinetic parameters for MurA were calculated by estimating the amount of P_i_ released from PEP. The *Km* and *Vmax* values of UDP-GlcNAc were measured at various concentrations of UDP-GlcNAc (0.2–3.0 mM) while keeping the PEP at a concentration of 1 mM. Likewise, the *Km* and *Vmax* values for PEP were measured at various concentrations (0.2–2.5 mM) while keeping UDP-GlcNAc at a concentration of 10 mM. The *Km* value of MurA for UDP-GlcNAc was found to be 2.048 mM and that for PEP was 0.5278 mM. The *Vmax* value for UDP-GlcNAc was 0.383 μM min^−1^ and for PEP was 0.173 μM min^−1^ (Table 1). Comparision of our data on MurA with those reported from a recent study^43^ on Mtb MurA showed *Km* and *Vmax* for UDP-GlcNAc to be similar, while for PEP, both the parameters (*Km* & *Vmax*) were found to be somewhat less, which could be attributed to variation in the reaction conditions in the assays.

MurB, reported to be dependent on K^+^ions for its activity^27^, was assayed at varying concentrations of KCl (1–20 mM). 10 mM was chosen as the final value since at this concentration, A_340_ decreased linearly (indicating oxidation of NADPH by MurB at a steady rate conditions), while at other concentrations, the oxidation was found to be either excessively fast or too slow (see Fig. S2A). The MurC to MurF enzymes are ATP-dependent ligases with stringent requirement for Mg^2+^ for activity. Here, reaction conditions were optimized first with MurC, it being the first of the four ligases, and its activity was tested at a range of concentrations of L-Alanine (0.2–2 mM) and MgCl_2_ (2–20 mM) while keeping ATP concentration fixed at 1 mM. Highest MurC activity was observed at 1 mM L-alanine and 10 mM MgCl_2_ (Fig. S2B). Subsequently, other ligases were examined for their respective substrates (D-Glutamate, *meso*-Diaminopimelic acid, and D-Alanine-D-Alanine) at 0.5 mM, 1 mM and 1.5 mM (based on the MurC optimization data range of concentration tested was narrow). Amino acids at a final concentration of 1 mM each and MgCl_2_ at 10 mM (data not shown) showed maximum activity for all four Mur ligases. The summary of reaction conditions for each enzyme (up to MurF), in a series of sequentially coupled assays is represented (Fig. S3).

### Electron Spray Ionization-Mass Spectrometry (ESI-MS) analysis of intermediates of the Mur pathway

**Figure 4:**
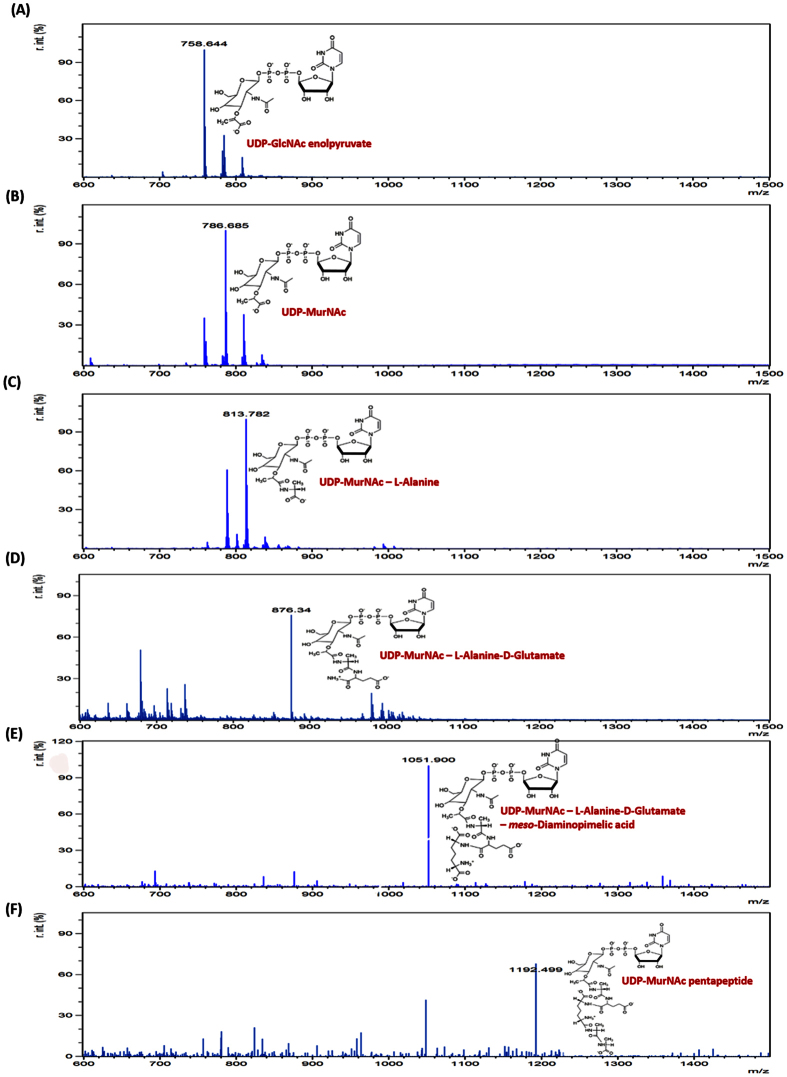
Electron Spray Ionisation-Mass Spectrometry (ESI-MS) analysis. Mass spectrum and chemical structures: **(A)** UDP-GlcNAc enolpyruvate, product of MurA. **(B)** UDP-MurNAc, Product of MurB. **(C)** UDP-MurNAc–L-Alanine, product of MurC. **(D)** UDP-MurNAc–L-Alanine–D-Glutamate, product of MurD. (**E)** UDP-MurNAc–L-Alanine–D-Glutamate–*meso*-Diaminopimelic acid, product of MurE. **(F)** UDP-MurNAc pentapeptide, product of MurF. The X-axis represents the mass to charge (m/z) ratio and the Y-axis represents the relative intensity of the peaks. The data was analyzed using mMASS^52^, an Open Source Mass Spectrometry tool to identify the products of the various assays.

The products formed after completion of each of the coupled enzyme assays were identified by ESI-MS analysis. The obtained mass spectrum validated the formation of product at the point of termination of each of the enzyme reaction (Fig. 4).

### Development and optimization of the one-pot assay for Mur enzymes

The principle of the one-pot assay is to reconstruct the Mur pathway *in vitro* in a single reaction where all the components (except for the nucleotide intermediates) are incubated together, leading to formation of the final product of the pathway (UDP-MurNAc pentapeptide). The concentration of assay components were further optimized to improve the sensitivity of the assay for screening of inhibitors. The components and parameters, which were optimized, are described below.

#### UDP-GlcNAc and PEP

**Figure 5:**
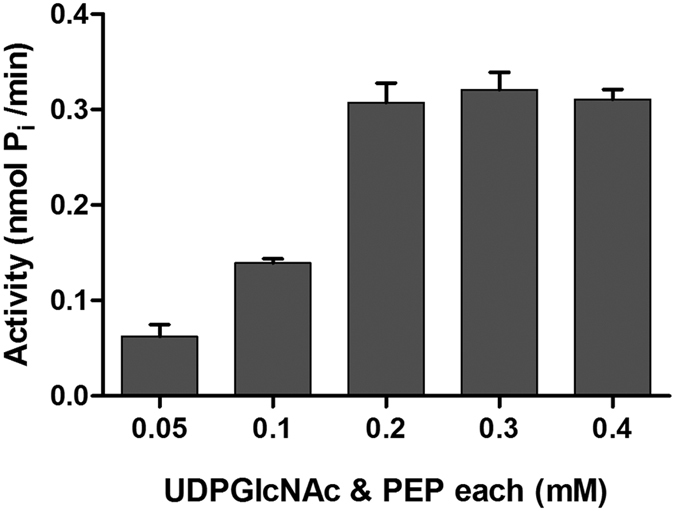
Effect of UDP-GlcNAc and PEP concentration on the one-pot assay. The X-axis represents the concentrations (mM) of the UDP-GlcNAc and PEP, each added in equal concentrations. The Y-axis represents net P_i_ (nmol P_i_/min). The Data depicted is mean ± S.E. values obtained from three independent experiments.

UDP-GlcNAc and PEP being the only substrates that were exogenously provided in the assay (the remaining intermediates were produced and consumed by recombinant Mur enzymes during enzymatic reactions) were optimized. A range of concentration of these initiating substrates (UDP-GlcNAc and PEP) was tested and the maximum activity was observed at 0.2 mM of each. Increasing the concentration of either substrate beyond 0.2 mM led to a decline in the P_i_ released in the one-pot assay which concurred with earlier reports where UDP-sugars at concentrations >0.2 mM are shown to significantly inhibit activity of Mur ligases^21^,^44^ (Fig. 5). Here we see that concentrations of UDP-GlcNAc and PEP used in the one-pot assay are much less than the values obtained when MurA enzyme activity was measured in a single reaction (Table 1). This interesting observation reflects upon how enzyme kinetics in a single reaction differs from that in a one-pot assay where extensive interdependence of reactions occur due to multi-enzyme activities.

#### ATP: One of the most critical components

In the one-pot assay, there were three potential sources of inorganic phosphate: (i) hydrolysis of ATP by Mur ligases during their respective synthetic reactions, (ii) non-enzymatic hydrolysis of ATP and (iii) un-coupled hydrolysis of ATP by Mur enzymes. Non-enzymatic hydrolysis of ATP, which could be attributed to the acidic pH of the detecting reagent used in measuring the P_i_, was normalized by measuring the P_i_ formed in the control reactions where all the constituents were incubated in the absence of Mur enzymes (data not shown). The un-coupled hydrolysis of ATP by Mur ligases has been reported elsewhere too^19^. We monitored un-coupled hydrolysis of ATP in control reactions where the initial substrate (UDP-GlcNAc) was not added to the one-pot and hence activity of any of the six Mur enzymes was not expected. In this control, amount of P_i_ detected were greater than those found in the control reactions showing non-enzymatic hydrolysis of ATP, hence suggesting that the additional P_i_ measured was from the un-coupled hydrolysis mediated by Mur enzymes.

**Figure 6:**
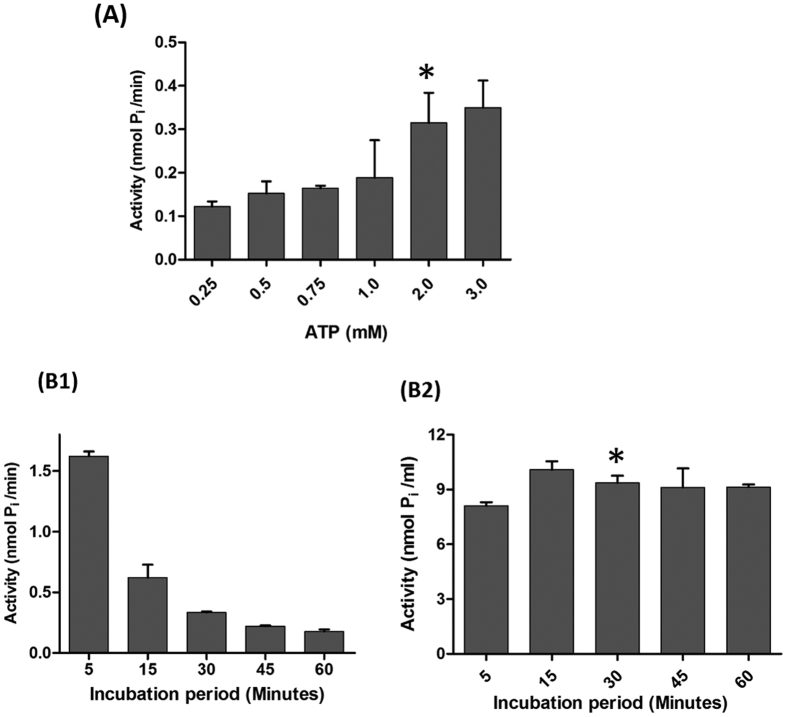
Optimization of the one-pot assay. (**A**) Effect of ATP concentration on the one-pot assay containing all the optimized constituents after 30 mins of incubation. The X-axis represents the concentration of ATP (0.25–3 mM) and the Y-axis represents net P_i_ per minute (nmol P_i_/min). **(B)** Activity of one-pot assay at different incubation periods. In B1, the X-axis represents incubation period (minutes) and the Y-axis represents net P_i_ per minute (nmol P_i_/min) and in B2, the X-axis represents incubation period (minutes) and Y-axis represents net P_i_ (nmol Pi/ml). The data depicted is mean ± S.E. values obtained from three independent experiments.

Another aspect of the assay which is highly dependent on the P_i_ concentration is the MurA enzymatic activity. In the presence of high levels of P_i_, MurA has been suggested to adopt an open, substrate-free form^41^ and therefore optimization of ATP concentration to be used in the one-pot assay is critical. While high concentration of ATP could lead to an open (and less active) conformation of MurA, lower concentration could compromise the overall activity of the Mur ligases. Testing ATP at concentrations from 0.25–3 mM, we found the activity to increase significantly up to 2 mM ATP, beyond which there was only a marginal rise in the net P_i_ production (Fig. 6A). Therefore, 2 mM ATP was used as the final concentration.

#### Incubation period

Finally, we were interested in determining the total incubation time as it is very often the case that products generated during the course of reaction in an assay impede the reaction by feedback inhibition of one or more enzymes. In the present context, MurA is known to be inhibited by two of the intermediates of the Mur pathway: UDP-MurNAc (product of MurB) and UDP-MurNAc pentapeptide (product of MurF)^42^. Considering these factors, the one-pot assay was carried out at various incubation periods and a significant increase in activity was observed by decreasing the incubation period from 5 hours to 5 minutes (data not shown). Though the rate of the reaction was found to be higher at five minutes (1.6226 nmol P_i_/min) of incubation than 30 minutes (0.3121 nmol P_i_/min), we kept 30 minutes as the final period as this duration was considered more conducive for handling large number of compounds for inhibitor studies, without compromising on the overall activity of the assay (Fig. 6B1,B2).

### Quality assessment of the one-pot assay

Eight replicates each of positive and control reactions were carried out to calculate the Z’-factor, a statistical parameter used for assessing the quality of an assay and its suitability for HTS^45^,^46^. We obtained a Z’ factor of 0.6, suggesting that the assay is amenable to HTS (Z’ values ≥0.5 are considered acceptable for HTS).

### Validation of the developed one-pot assay as an efficient screening tool for identifying multi-target inhibitors

After successfully developing and validating the one-pot assay for the reconstruction of the Mur pathway in Mtb, we were interested in establishing the sensitivity of the assay towards inhibitors and in evaluating Mur ligases (MurC-MurF) as multiple drug targets. While studying the effect of inhibitors on the assay, we used purified MBP as a control since MurD and MurF enzymes were purified with MBP tag. To further ascertain that MBP tag does not interfere with binding of inhibitor to the enzyme, we evaluated the compounds on both His- and MBP-tagged MurD and MurF and found similar inhibition profile (data not shown). His-tagged MurD and MurF proteins were not included in this study since their yield as soluble protein was very poor.

A set of 18 available compounds classified as Furan-based benzene mono- and dicarboxylic acid derivatives and Benzene-1,3-dicarboxylic acid 2,5-dimethylpyrrole derivatives^33^,^47^,^48^, previously tested for their inhibitory effects on Mur ligases from *E.coli* were included in this study (Tables S2 and S3). These compounds belong to a novel class of glutamic acid surrogates-benzene mono and dicarboxylic acid derivatives with an incorporated furan or 2,5-dimethylpyrrole moiety. They were primarily designed to improve the physicochemical properties as well as binding properties of the D-Glu inhibitors known to possess dual MurD-MurE inhibition activies^47^. Some members of this test set were shown to possess promising multiple MurC-MurF *E. coli* inhibitory activity as well as antibacterial activity^33^,^48^. In addition, selected compounds were also studied in steady-state kinetic studies and as such represent a suitable molecular tool for validating the developed Mtb Mur one-pot assay.

Out of 18 compounds assayed, compounds **3** & **4** from the furan-based benzene mono carboxylic acid derivatives demonstrated significant inhibition of the one-pot assay with an IC_50_ value of 19.6 μM and 8.25 μM, respectively, while the remaining compounds did not show significant inhibition. Next, we investigated the effect of these two inhibitors on each of the Mur enzymes and interestingly, differential inhibition of enzymes was observed (Table 2). While, compound **3** showed marginal inhibition of MurA, MurC and MurD enzymes and no inhibition of MurB, compound **4** did not inhibit MurA, MurB at all and showed marginal inhibition of MurC, MurD enzymes. Significant inhibition of MurE and MurF was observed by compound **4** with the IC_50_ of 17 μM and 25.8 μM, respectively. Targets for compound **3** were also MurE and MurF but with high IC_50_ of 61.6 μM and 45.4 μM, respectively. These findings are in concurrence with the previous study^48^ where compound **3** & **4** inhibited MurE and MurF from *E.coli* with IC_50_ values of 11–16 μM^48^. The higher IC_50_ values for Mtb Mur ligases could be attributed to significant variation in their primary sequences and known low sequence homology of Mur ligases from different bacterial species leading to local changes in their structural topology^12^. Lower IC_50_ values of one-pot assay in comparison to IC_50_ values of individual MurE and MurF assays could be attributed to the cumulative inhibition on multiple enzymes of the pathway.

D-Cycloserine, a clinically approved drug^49^,^50^ used in the treatment of tuberculosis where its established target is D-Alanine-D-Alanine ligase (*ddl*) was tested for inhibition of the Mur pathway using the one-pot assay. Despite the fact that the four ligases in the one-pot assay are different in their structural topology and function from *ddl*, we decided to investigate if this drug molecule might also inhibit activity of one or more of the Mur ligases. Interestingly, D-Cycloserine showed inhibition of the Mur pathway, albeit at a higher concentration (IC_50_ = 74 μM). Following further investigations, MurF (IC_50_ = 67.1 μM) emerged as the likely target of D-Cycloserine. MurE was also inhibited but its IC_50_ was higher (IC_50_ = 97 μM) (Table 2). The ESI-MS analysis of D-Cycloserine activity in one-pot assay shows inhibition of final product formation and hence further corroborates that D-Cycloserine is an inhibitor of Mur Pathway (Fig. S5). This observation is significant, as D-Cycloserine has not previously been demonstrated to be an inhibitor of Mur enzymes from Mtb, pointing to an additional mechanism of action of this important antibacterial drug molecule.

To conclude, to the best of our knowledge this is the first study, where a convenient one-pot assay for the reconstruction of the entire Mur pathway (MurA-MurF) in *Mycobacterium tuberculosis* is developed, optimized and validated with a demonstrated potential to screen inhibitors that target multiple enzymes in a single reaction. This being an assay that does not mandate the addition of intermediates of the pathway adds to its potential for cost-effective high throughput screening in much-needed antibacterial drug design efforts. Known furan-based monocarboxylic compounds **3** & **4** were used to validate the assay and thus we were able to show that these compounds could provide a starting point for further optimization towards molecules with favourable enzyme inhibition properties of the Mtb Mur pathway and anti-Mycobacterial activity. In addition, the inhibitory activity of the known drug molecule D-Cycloserine on the Mur pathway, particularly on the MurF enzyme was established thus suggesting an additional mechanism of this drug molecule. Further studies to gain mechanistic insight into the inhibition of Mtb Mur ligases and developing these compounds as potential drug candidates are planned. In summary, the present findings validate that the developed one-pot assay could be used for identifying novel Mur pathway inhibitors that could potentially be used in multi-targeted therapy.

## Materials and Methods

### Bacterial strains, chemicals and reagents

*Escherichia coli* DH5α was used for the general cloning procedures and *E.coli* BL21 (DE3), C41 (DE3) and Rosetta-gami™ (DE3) pLysS were used for expression studies. The restriction endonucleases, DNA-modifying enzymes, amylose resin were purchased from New England Biolabs (USA). Primers and analytical grade chemicals were purchased from Sigma-Aldrich. Ni-NTA agarose and other PCR purification/Plasmid isolation kits were obtained from Qiagen (Germany). Phenylmethanesulfonylfluoride (PMSF) and other chemicals were purchased from Himedia Laboratories (India). P_i_ColorLock Gold reagent was purchased from Innova Biosciences, UK. The vectors pMAL-c2X and pET28a were kindly given by Dr.Vinay K. Nandicoori (National Institute of Immunology, New Delhi) and Dr.S. Ramachandran (Institute of Genomics and Integrative Biology, New Delhi), respectively.

### Cloning, expression and purification of Mur enzymes

The *murA*-*murF* genes were PCR amplified from Mtb H37Rv genomic DNA using the primers listed in Table S1 and Phusion High Fidelity DNA polymerase (NEB, USA). The amplicons were cloned into pET28a vector and were confirmed by DNA sequencing. The pET28a plasmid constructs were transformed into *E. coli C41* (DE3) (Novagen). Fresh transformants were grown in 500 ml of LB medium containing 50 μg/ml kanamycin to a cell density of ~*A*_600_ of 0.6. The cells were induced with 0.1 mM IPTG and grown for 18 h at 25 °C. *His-tagged* proteins so over-expressed, were purified following the manufacturer’s (Qiagen, Germany) recommendations. The cells were harvested and resuspended in 30 ml of lysis buffer (20 mM Tris-HCl, pH 8.0, 500 mM NaCl, 10 mM imidazole, 1 mM PMSF) and lysed by sonication, the supernatant containing the soluble fraction was clarified by centrifugation at 14, 500 × g for 30 min at 4 °C and loaded to a pre-equilibrated (1 ml bed volume) Ni–NTA affinity column. After washing the column with 30 bed volumes each of Wash Buffer I (20 mM Tris- HCl, pH 8.0, 500 mM NaCl and 20 mM imidazole) and Wash buffer II (20 mM Tris-HCl, pH 8.0, 500 mM NaCl and 40 mM imidazole), the bound protein was eluted in Elution buffer (20 mM Tris-HCl, pH 8.0, 500 mM NaCl and 200 mM imidazole). The eluted proteins were dialyzed against the storage buffer (20 mM Tris-HCl, pH 8.0, 150 mM NaCl, 5 mM β-mercaptoethanol, and 10% glycerol). The purified proteins were quantified using the Bradford method^51^ and stored at −80 °C.

MurD and MurF could not be obtained in soluble form when expressed in the pET28a and were hence cloned in the pMALc2X vector and transformed into *E.coli* Rosetta-gami™ (DE3) pLysS. Fresh transformants were grown in 500 ml of LB medium containing 100 μg/ml ampicillin to a cell density of ~*A*_600_ of 0.6. The cells were induced with 0.1 mM IPTG and grown for 18 h at 16 °C. The *maltose binding protein (MBP) tagged* proteins were purified following manufacturer’s recommendations (NEB). The cells were harvested and lysed by sonication in 30 ml of column buffer (20 mM Tris-HCl, pH 8.0, 300 mM NaCl, 1 mM EDTA and 5 mM β-mercaptoethanol). The supernatant containing the soluble fraction was purified by affinity chromatography using amylose resin. After washing the column with 100 bed volumes of column buffer, the bound protein was eluted in column buffer containing 10 mM Maltose. The eluted proteins were dialyzed against storage buffer (20 mM Tris-HCl, pH 8.0, 150 mM NaCl, 5 mM β-mercaptoethanol, and 10% glycerol). The purified proteins were quantified using the Bradford method^51^ and stored at −80 °C.

### Mur enzyme assays

The assays for all the Mur enzymes (MurA-MurF) were carried out as previously described^20^,^27^,^34^ with slight modifications. Different concentrations of enzymes and substrates, various buffers at different pH and the effect of salts were tested. In reactions where MBP-tagged enzymes (MurD and MurF) were used, purified MBP (1 μg) was included in the control reactions.

#### MurA assay

The MurA reaction mixture (50 μl) contained 50 mM Bis-tris propane buffer (pH 7.0), 0.2 mM UDP-GlcNAc, 0.2 mM PEP and 125 nM of MurA enzyme. The reaction was performed in a micro-titer plate at 37 °C for 30 minutes and terminated by the addition of P_i_ Colorlock™ Assay reagent. Absorbance was measured at 630 nm and the amount of P_i_ released was calculated from the standard phosphate curve. Care was taken to correct absorbance values for background P_i_ hence throughout; net P_i_ is expressed as product. The control reactions contained all the components except MurA. The net P_i_ was calculated by subtracting total Pi in the control reaction from the total Pi after the completion of the assay. To get a large assay window and to avoid saturation of P_i_, optimization of assays were carried out at substrate concentrations so as not to exceed the assay signal beyond 2.0 absorbance units as recommended by the manufacturer of PiColorlock™ Assay kit.

Kinetic parameters for MurA were calculated by estimating the amount of P_i_ released from PEP. The *Km* and *Vmax* values of UDP-GlcNAc were measured at various concentrations of UDP-GlcNAc (0.2–3.0 mM) while keeping PEP at a concentration of 1 mM. Likewise, the *Km* and *Vmax* values for PEP were measured at various concentrations (0.2–2.5 mM) while keeping UDP-GlcNAc at a concentration of 10 mM. The control reaction contained all the components except MurA.

#### MurB assay

To assay MurB activity, the MurA reaction after 30 mins of incubation was supplemented with 10 mM KCl, 0.2 mM NADPH and 60 nM of MurB enzyme in a total volume of 100 μl. The absorbance at 340 nm was monitored over 5 min to calculate the rate of NADPH oxidation. The control reaction contained all the components except MurB.

#### Assay for Mur Ligases

Each Mur ligase reaction was performed at 37 °C with an incubation period of 1 h. To measure the activity of MurC, the completed MurB reaction was supplemented with 1 mM L-Alanine, 10 mM MgCl_2_ and 1 mM ATP. MurD-F ligase activities were performed sequentially, supplementing the prior reaction with 1 mM each of the required substrate (D-Glutamate, *meso*-Diaminopimelic acid and D -Alanine-D-Alanine, respectively). The control reaction contained all the components except the enzyme that was being tested (e.g. the control reaction for MurC assay contained all the components except MurC). For MurD and MurF reaction, purified MBP at 1 μg concentration was added to the reaction mixture instead of the enzymes. Estimation of net P_i_ was done as previously described in the MurA assay.

### Development of a one-pot assay for the Mur pathway

**Figure 7:**
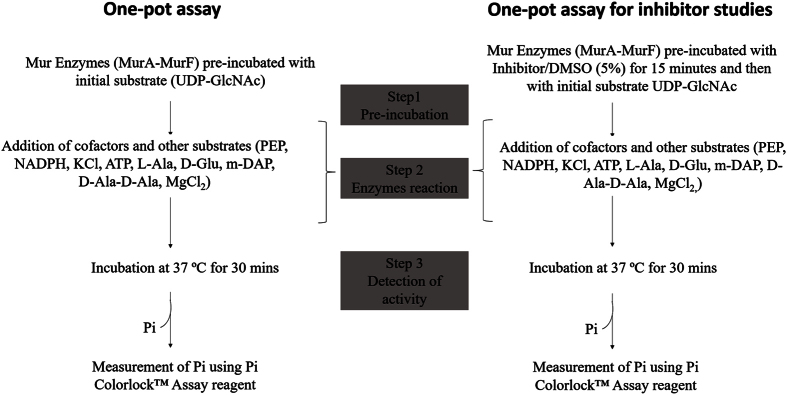
Steps outlining the development of the one-pot assay for the *in vitro* reconstruction of the Mur pathway.

In the developed one-pot assay, all the constituents were incubated together than in a sequential manner (Fig. 7). The final reaction mixture in a total volume of 100 μl contained the following: Bis-tris propane (50 mM, pH 7.0), L-Alanine, D-Glutamate, *meso*-Diaminopimelate, D-Alanine-D-Alanine (1 mM each), UDP-n-acetylglucosamine (0.2 mM), PEP (0.2 mM), NADPH (0.2 mM), KCl (10 mM), MgCl_2_ (10 mM), ATP (2 mM), MurA (125 nM), MurB (60 nM), MurC (100 nM), MurD (93 nM), MurE (135 nM) and MurF (100 nM) and incubated at 37 °C. Purified MBP at 1 μg concentration was added in place of MurD and MurF enzymes in the control reaction (Fig. S4). The control reaction contained all the components except UDP-GlcNAc. The net P_i_ released was calculated as described earlier.

### Electron Spray Ionization-Mass Spectrometry (ESI-MS) analysis

After the completion of enzyme assays (MurA assay, subsequent successive coupled assays and the one-pot assay), the reaction mixture was filtered through Amicon ultra-0.5 ml centrifugal filters to eliminate enzyme(s) and the filtrate obtained was submitted for ESI-MS analysis at AIRF, JNU, New Delhi. Separation was done using ACQUITY UPLC^®^ BEH C18 1.7 μm column (Waters, USA). Flow rate was maintained at 0.2 ml/min, the injection volume was 5 μl, and the ESI-MS spectra were obtained using the positive and negative ion mode. Mass spectrometry detection was performed with the ESI set at a capillary voltage of 2.72 kV and capillary temperature of 110 °C. Full scan spectra were obtained in the range of 600–1500 m/z. The data was analyzed using mMASS^52^, an Open Source Mass Spectrometry tool.

### Inhibitory effect of D-Cycloserine and a series of mono- and dicarboxylic acid derivatives

#### Composition of one-pot assay for inhibitor studies

The final reaction mixture in a total volume of 100 μl contained the following: Bis-tris propane (50 mM, pH 7.0), L-Alanine, D-Glutamate, *meso*-Diaminopimelate, D-Alanine-D-Alanine (0.5 mM each), UDP-n-acetylglucosamine (0.2 mM), PEP (0.2 mM), NADPH (0.2 mM), KCl (10 mM), MgCl_2_ (10 mM), ATP (1.5 mM), MurA (55 nM), MurB (30 nM), MurC (45 nM), MurD (45 nM), MurE (45 nM) and MurF (30 nM) and incubated at 37 °C.

Tested compounds at various concentrations (5–100 μM) were pre-incubated with enzymes for 15 mins, followed by the addition of remaining constituents of the assay. The final reaction mixture was incubated for 30 mins at 37 °C. To assess specific inhibition of Mur ligases, each of the ligase was pre-incubated with inhibitors separately and then added to the one-pot reaction mixture. The net P_i_ released was calculated as described earlier. In the ‘no drug’ control reaction, the organic solvent (DMSO) used in dissolving inhibitory compounds was added at 5% concentration to the one-pot assay, while Isoniazid, a standard anti-TB drug with no known effect on Mur activity, was inculded as another control to ascertain the specificity of the tested inhibitors in the study.

## Additional Information

**How to cite this article**: Eniyan, K. *et al.* Development of a one-pot assay for screening and identification of Mur pathway inhibitors in *Mycobacterium tuberculosis. Sci. Rep.* **6**, 35134; doi: 10.1038/srep35134 (2016).

## Supplementary Material

Supplementary Information

## Figures and Tables

**Table 1:** Determined kinetic parameters of Mtb MurA.

	Substrate	Km (mM)	Vmax (μM min^−1^)
Mtb MurA	UDP-GlcNAc	2.048	0.383
	PEP	0.5278	0.173

**Table 2:**
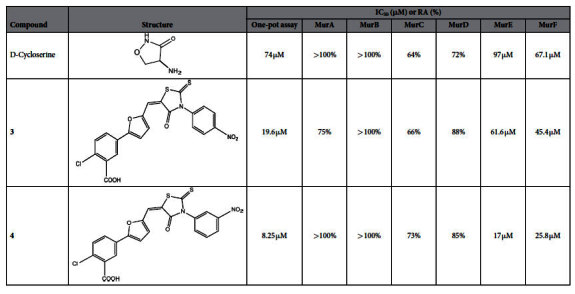
Results of the inhibition of one-pot assay by D-Cycloserine, Compound 3 and Compound 4 against Mur Enzymes from Mtb.

Residual activities (RAs) were calculated with respect to ‘no drug’ control assays (containing 5% DMSO) at 100 μM concentration. The IC_50_ was determined by measuring the activity at six different compound concentrations (5, 10, 25, 50, 75, 100 μM) and represents the concentration for which the RA was 50%.
