# Supplementary material for: Development of a one-pot assay for screening and identification of Mur pathway inhibitors in Mycobacterium tuberculosis
Source: Sci Rep. 2016 Oct 13;6:35134. doi: 10.1038/srep35134 (PMC5062083; doi:10.1038/srep35134)
Supplement: Supplementary Information [file srep35134-s1.pdf]

Development of a one-pot assay for screening and identification of Mur pathway inhibitors in *Mycobacterium tuberculosis*

Kandasamy Eniyan[1], Anuradha Kumar[2], Geetha Vani Rayasam[2], Andrej Perdih[3] and Urmi Bajpai[1*]

[1]Department of Biomedical Science, Acharya Narendra Dev College, University of Delhi, New Delhi 110019, India

[2]Open Source Drug Discovery Unit, CSIR, New Delhi 110002, India

[3]National Institute of Chemistry, Hajdrihova 19, 1001 Ljubljana, Slovenia

***Corressponding author**
Urmi Bajpai, PhD
Department of Biomedical Science,
Acharya Narendra Dev College,
University of Delhi
New Delhi 110019, India
Email: urmibajpai@andc.du.ac.in
Ph. No. 011-26412547
Fax: 011-26294540

Supplementary information

Table S1. Primers used in amplification of Mur enzymes

Primer name	Primer DNA sequence	Restriction enzymes
MurA F	5'-ACCCTTGAATTCACTGACCGG-3'	*EcoRI*
MurA R	5'-AGGTAAGCTTCGCCTAACAGC-3'	*HindIII*
MurB F	5'-ATTAGAATTCATGAAACGGAGCGGTGTC-3'	*EcoRI*
MurB R	5'-GCCGCGAAAAAGCTTCTACAAC-3'	*Hind III*
MurC F	5'-ATTAGGATCCGTGAGCACCGAGCAGTTG-3'	*BamHI*
MurC R	5'-TCTAAGCTTCCTCGTTGTGTTCC-3'	*Hind III*
MurD F	5'-CGCGGATCCGACGTGCTTGACCCTCTG-3'	*BamHI*
MurD R	5'-CGCAAGCTTCTACCGGATCACCGCGCG-3'	*Hind III*
MurE F	5'-CGCGGATCCGTGTCATCGCTGGCCCGAG-3'	*BamHI*
MurE R	5'-CGCAAGCTTTCATGCGCGCCGCTCGAG-3'	*Hind III*
MurF F	5'-CGCGGATCCATGATCGAGCTGACCGTCGCG-3'	*BamHI*
MurF R	5'-CGCAAGCTTCATGGGCGCACACTCCC-3'	*Hind III*

The underlined bases indicate the restriction sites.

Table S2. Results of the one-pot assay of the furan-based mono and dicarboxylic acid molecules against Mur Enzymes from *M.tuberculosis*.

**Compound**	**Structure**	**Percentage inhibition (%)**	**Compound**	**Structure**	**Percentage inhibition (%)**
**1**	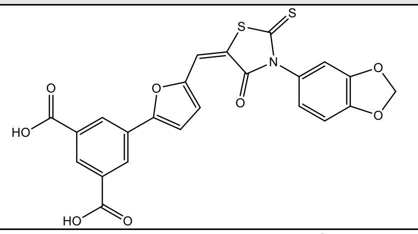	16	**6**	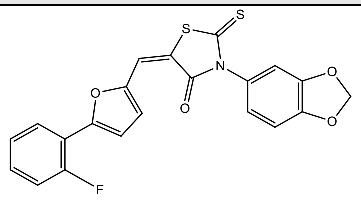	16
**2**	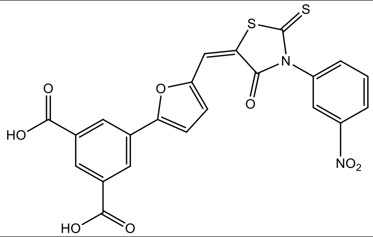	18	**7**	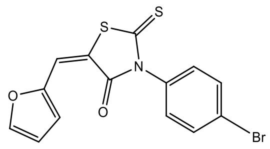	10
**3**	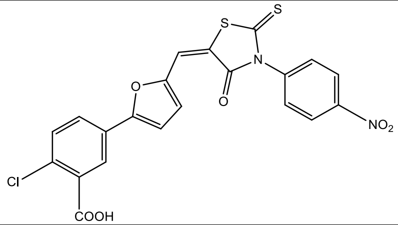	97	**8**	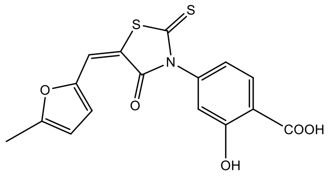	21
4	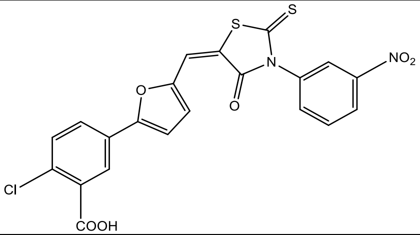	93	**9**	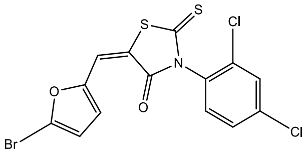	**-1**
**5**	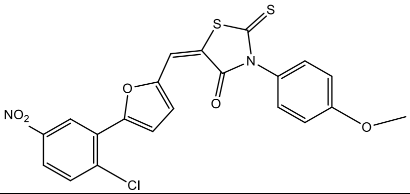	10	**10**	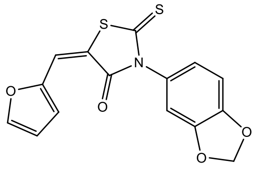	28

Inhibition in % were determined at 100 μM concentration of the investigated compound.

Table S3. Results of the one-pot assay of the selected benzene-1,3-dicarboxylic acid 2,5-dimethylpyrrole derivatives against Mur Enzymes of *M.tuberculosis*.

Compound	Structure	Percentage inhibition	Compound	Structure	Percentage inhibition
**A**	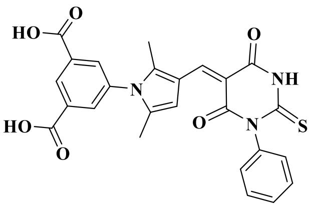	6	**E**	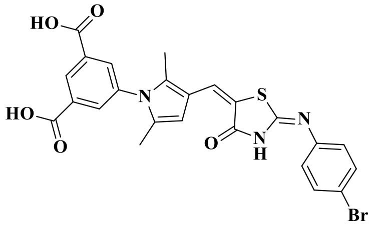	2
**B**	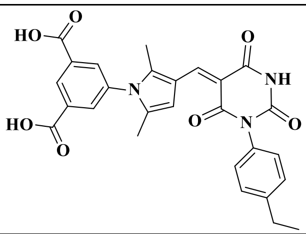	16	**F**	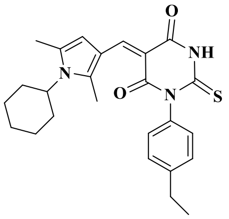	9
**C**	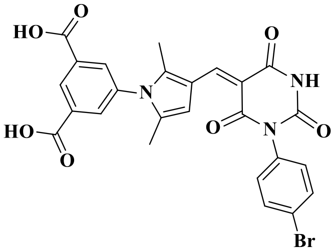	3	**G**	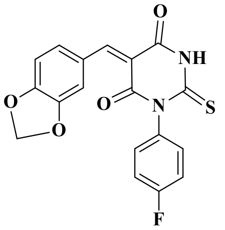	10
**D**	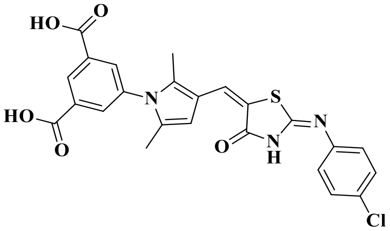	10	**H**	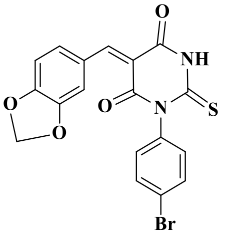	15

Inhibition in % were determined at 250 µM concentration of the investigated compound.

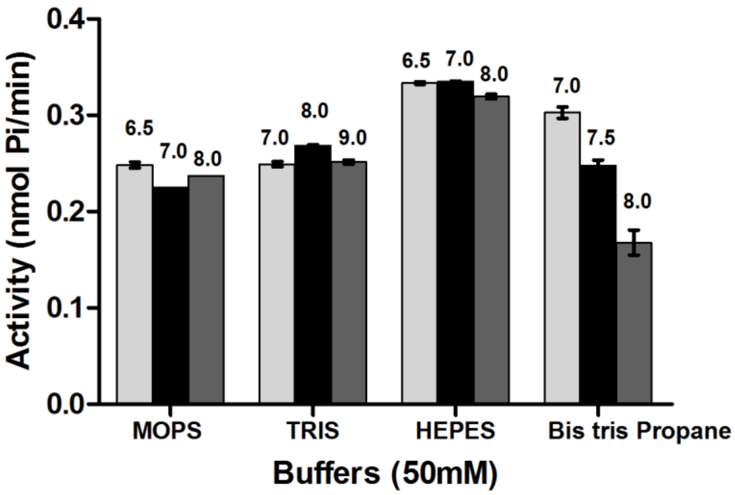

Figure S1. Effect of various buffers on the UDP-N-acetylglucosamine enolpyruvyl transferase activity of MurA enzyme. The pH of the buffers tested are mentioned above the bar. Net Pi released in enzymatic reactions was determined and data depicted is mean ± S.E. values obtained from three independent experiments.

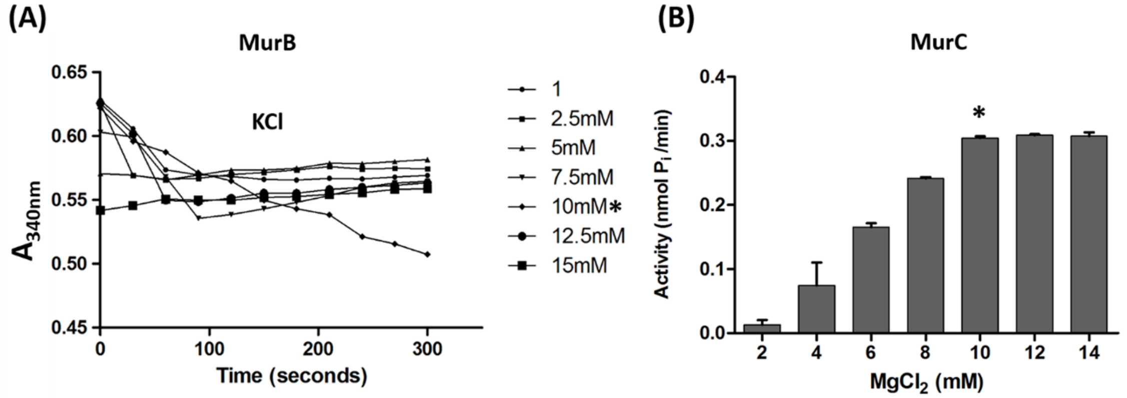

Figure S2. Optimization of K^+ and Mg^{2+} on MurB and MurC (first of the four ligases) (A) Effect of K^+ on the activity of MurB enzyme. X-axis represents incubation time (seconds) and Y-axis represents absorbance at 340nm and (B) Effect of Mg^{2+} on the ATPase activity of MurC enzyme. X-axis represents the $MgCl_2$ at various concentrations (mM) and Y-axis represents the amount of Pi released (nmol/min). Net Pi released in enzymatic reactions was determined and data depicted is mean $\pm$ S.E. values obtained from three independent experiments.

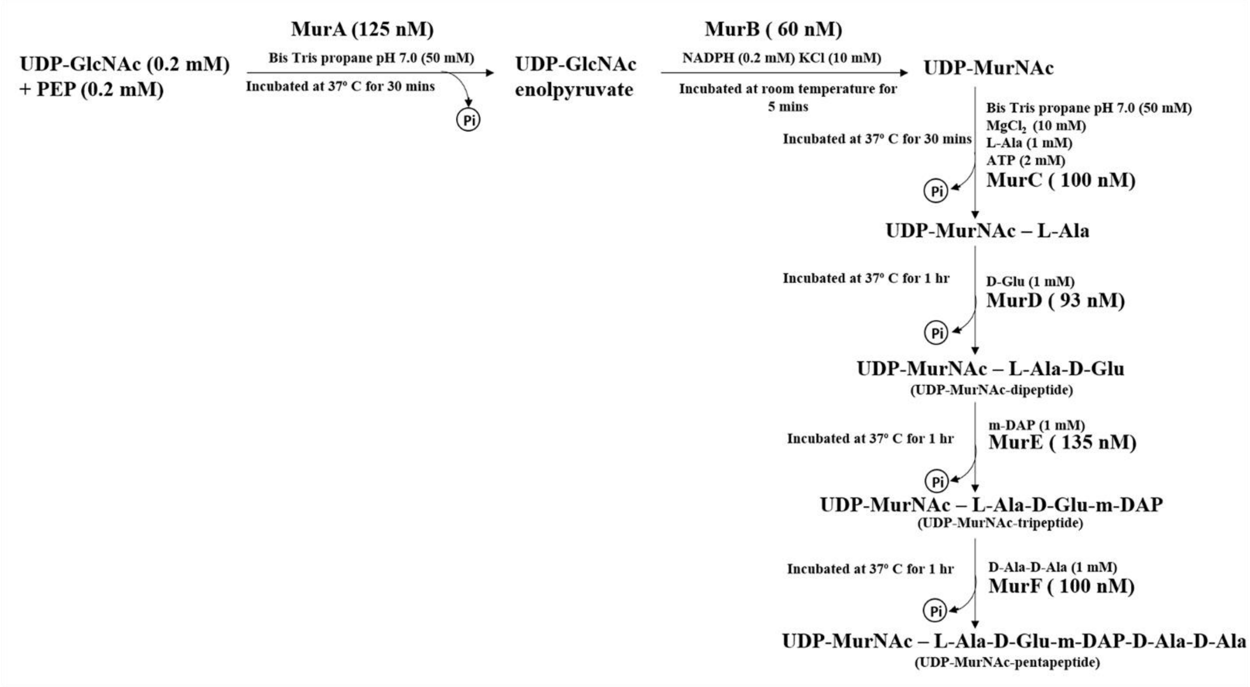

Figure S3. Schematic representation of the sequential coupled assays.

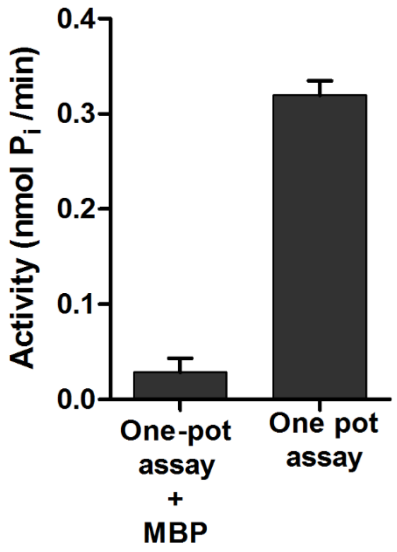

Figure S4. Effect of purified MBP in the one-pot assay. Purified MBP at 1μg concentration was added in place of MurD and MurF enzymes in the reaction mixture to demonstrate that the observed activity was specific to MurD and MurF. X-axis represents MBP added in one-pot assay and Y-axis represents net P_i (nmol P_i /min)

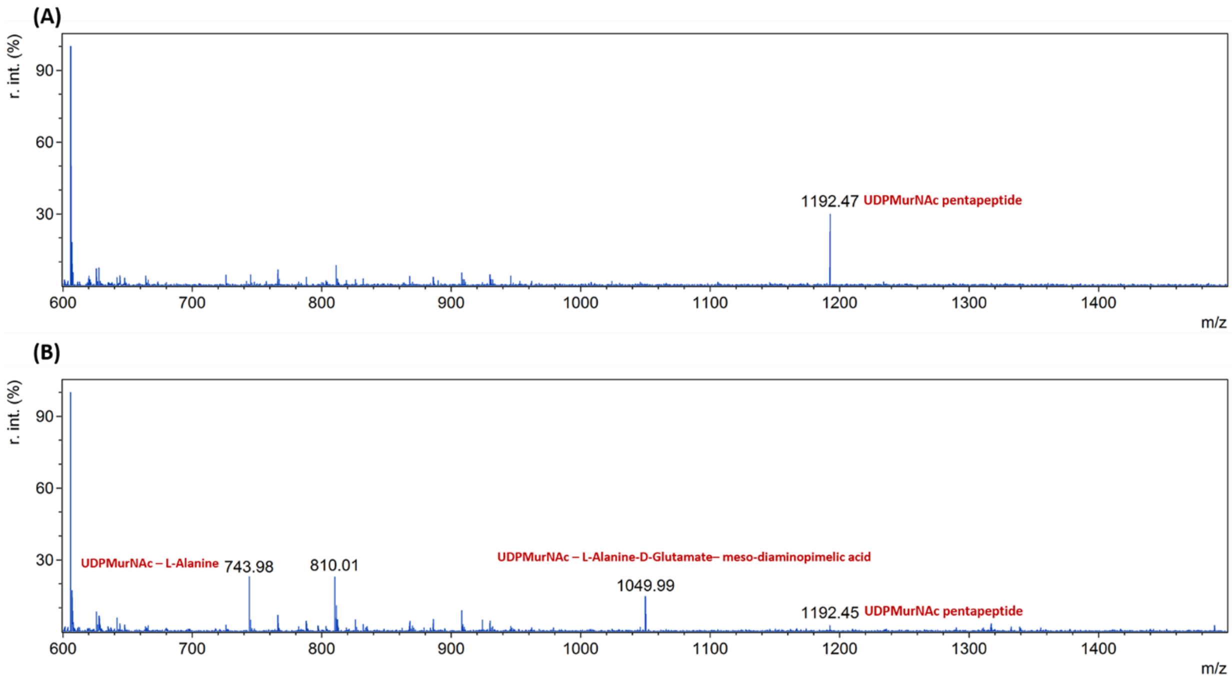

Figure S5. Electron Spray Ionization-Mass Spectrometry analysis of D-Cycloserine activity in one-pot assay. (A) ESI-MS analysis of one-pot assay without D-Cycloserine**. (B)** ESI-MS analysis of one-pot assay with D-Cycloserine**.**
